# A First-of-Its-Kind Case of Annular Lichenoid Dermatitis of Youth (ALDY) From Kuwait

**DOI:** 10.7759/cureus.104999

**Published:** 2026-03-10

**Authors:** Danah Alkandari, Hasan KH Ashkanani, Mohammd Aladwani, Heba Elmewafy, Ghadeer Alalawi, Talal Almutairi

**Affiliations:** 1 Dermatology, Kuwait Institute for Medical Specializations, Kuwait City, KWT; 2 Dermatology, Amiri Hospital, Kuwait City, KWT; 3 Dermatology, Farwaniya Hospital, Kuwait City, KWT; 4 Dermatology, Al Adan Hospital, Hadiya, KWT

**Keywords:** aldy, annular lichenoid dermatitis of youth, dermatology, lichenoid interface dermatitis, mycosis fungoides

## Abstract

Annular lichenoid dermatitis of youth (ALDY) is a rare, benign lichenoid dermatosis characterized by annular lesions and distinctive histopathologic features, including lichenoid interface dermatitis with prominent basal keratinocyte apoptosis localized to the tips of rete ridges. Although originally described in children, adult-onset cases are increasingly recognized. We report the first case of ALDY in Kuwait (to the best of our knowledge) in a 37-year-old Kuwaiti man with a four-year history of an asymptomatic erythematous patch on the left flank. Histopathology showed classic quadrangular rete ridge changes with a benign lymphocytic infiltrate and a near-equal CD4:CD8 ratio. Recognition of ALDY is essential to avoid misdiagnosis, particularly with early mycosis fungoides, and to ensure appropriate conservative management.

## Introduction

Lichenoid dermatoses are a wide spectrum of dermatological disorders characterized by vacuolar alteration and apoptotic keratinocytes in the basal layer of the epidermis, with a band-like lymphohistiocytic infiltrate obscuring the dermoepidermal junction [1]. These histological changes result in clinical manifestations including flat-topped violaceous papules, annular or linear plaques, papulovesicles, and erythematous macules [2]. However, in a 2003 Italian study, a series of young patients presented with peculiar skin changes characterized by annular patches and persistent erythematous macules, often localized to the groin and flanks [1]. The differential diagnosis of this condition included plaque-stage mycosis fungoides (MF), annular erythema, and inflammatory morphea; however, these entities were excluded based on distinctive histopathologic findings, namely, a superficial lichenoid dermatitis with massive apoptosis of keratinocytes localized to the tips of the rete ridges [1]. This clinicopathologic entity was subsequently termed annular lichenoid dermatitis of youth (ALDY).

ALDY is typically characterized by solitary or multiple plaques or erythematous macules that evolve into well-defined annular lesions with raised borders and a hypopigmented center [3]. In older lesions, the borders may become hyperpigmented in a continuous or segmented pattern [4]. Lesions most commonly involve the trunk, particularly the flanks, inguinal region, and abdomen, with a predilection for flexural areas including the axillae, inframammary regions, periumbilical area, and neck [5]. ALDY is generally asymptomatic, although mild pruritus may occur during the development of new lesions [6]. Although the condition is named for its predominance in pediatric and young adult populations, reported cases span a wide age range from 2 to 79 years, with a mean age of 14.7 years, and only 67 cases have been documented in the literature to date [3].

## Case presentation

**Figure 1:**
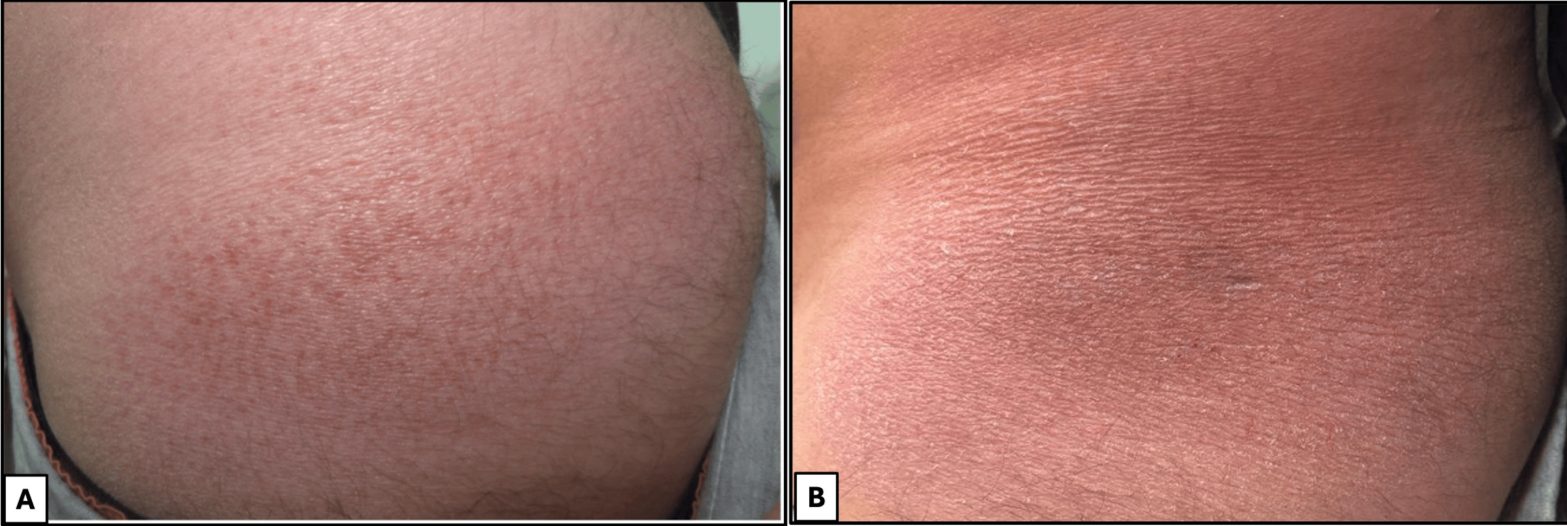
Clinical presentation and biopsy site of annular lichenoid dermatitis of youth (ALDY) on the left flank (A) A solitary, ill-defined, non-scaly, erythematous patch measuring 10 × 5 cm localized to the left flank. The lesion exhibits the characteristic annular configuration with a hypopigmented central zone, four years post-onset. (B) Clinical photograph of the left flank following a 4-mm punch biopsy, performed to evaluate the clinical differential diagnoses including mycosis fungoides, sarcoidosis, and eczematous dermatitis.

We present a case of a 37-year-old Kuwaiti man with no significant past medical history who was evaluated in the dermatology clinic in July 2025 for a progressive, asymptomatic skin rash on his left flank of four years’ duration. Physical examination revealed a solitary, ill-defined, non-scaly erythematous patch measuring 10 × 5 cm (Figure 1A). The patient’s initial presentation was delayed due to the asymptomatic nature of the lesion, and he expressed reluctance toward biopsy at the initial consultation. Examination of the scalp, nails, and mucous membranes was unremarkable.

Baseline laboratory investigations, including complete blood count, comprehensive metabolic panel, thyroid function tests, lipid profile, glycated hemoglobin (HbA1c), erythrocyte sedimentation rate, and C-reactive protein, were all within normal limits. A punch biopsy was performed to evaluate the clinical differential diagnoses of patch-stage mycosis fungoides, cutaneous sarcoidosis, and eczematous dermatitis (Figure 1B).

**Figure 2:**
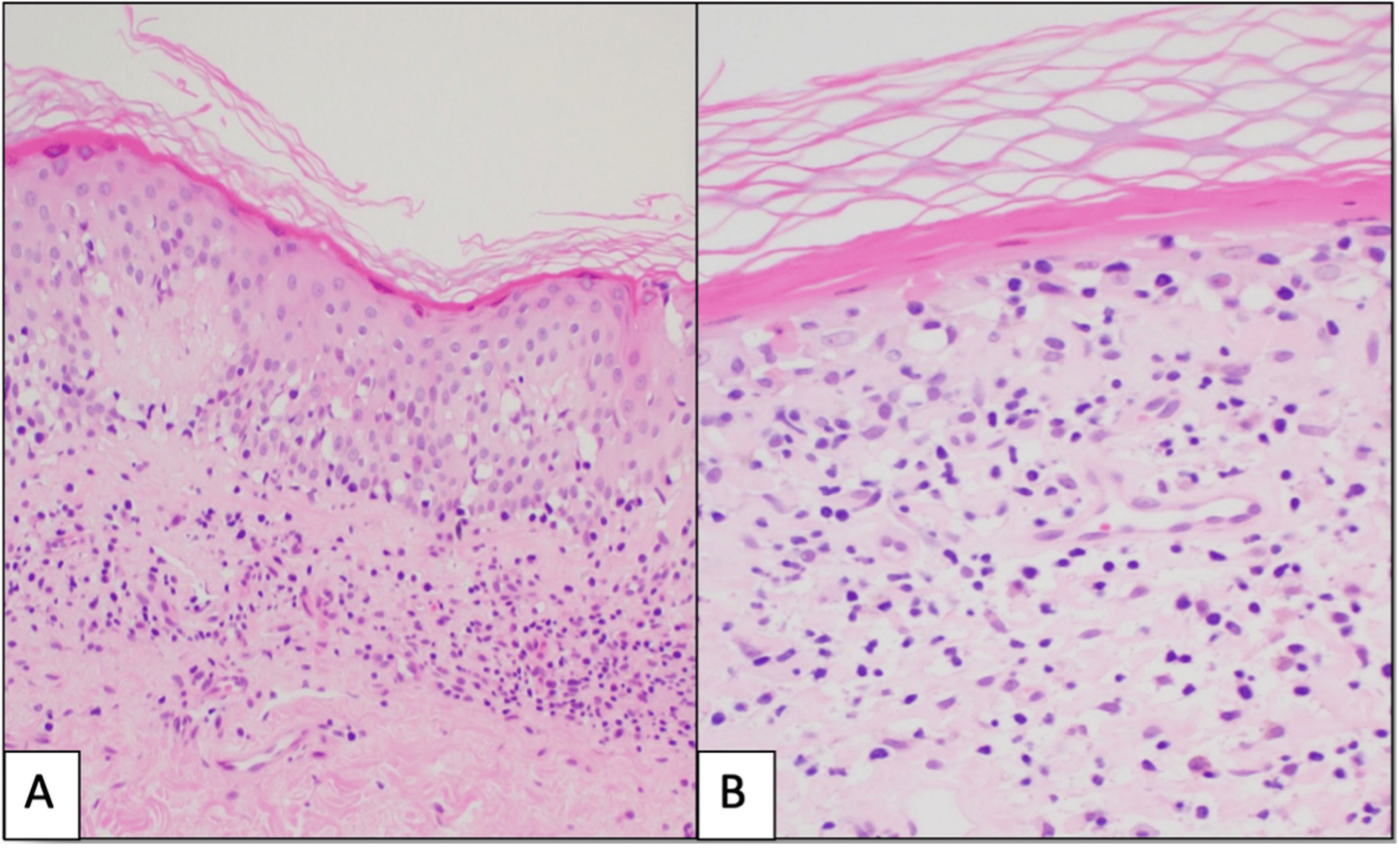
H&E sections showing (A) lichenoid interface dermatitis with square-shaped rete ridges and (B) flattening of the epidermis with apoptotic keratinocytes and dermal melanophages Scanning magnification demonstrates a band-like lichenoid lymphocytic infiltrate obscuring the dermoepidermal junction. There is irregular acanthosis with distinctive “squaring” of the rete ridges (H&E, original magnification x100) (A). Higher magnification highlights massive apoptosis of basal keratinocytes strictly confined to the tips of the rete ridges, imparting a quadrangular morphology. Vacuolar interface changes and scattered dermal melanophages are present, with no significant epidermotropism or atypical lymphocytes (H&E, original magnification x200) (B).

Histopathologic examination demonstrated a dense, band-like lymphocytic infiltrate within the papillary dermis, accompanied by vacuolar interface changes. Notably, there was prominent apoptosis of keratinocytes strictly confined to the tips of the rete ridges, imparting a distinctive quadrangular morphology (Figures 2A, 2B). The epidermis showed focal flattening without significant spongiosis or hyperkeratosis. Immunohistochemical staining revealed a near-equal CD4:CD8 ratio within the lymphocytic infiltrate, supporting a benign inflammatory process.

The constellation of clinical and histopathologic findings confirmed a diagnosis of annular lichenoid dermatitis of youth. First-line treatment was initiated with a one-month course of combination therapy using a high-potency topical corticosteroid and a topical calcineurin inhibitor, resulting in partial clinical improvement.

## Discussion

Clinical and histopathologic features of ALDY

Annular lichenoid dermatitis of youth is a distinctive lichenoid dermatosis first described by Annessi et al. in 2003 as a peculiar interface dermatitis occurring in young patients (median age ~10 years) with annular lesions [1]. Clinically, ALDY presents as persistent, asymptomatic round or oval patches with sharply demarcated annular configurations. Lesions characteristically have a red-brown or violaceous elevated border and a centrally hypopigmented or slightly atrophic-appearing zone [7]. The patches typically lack scale (helping distinguish them from eczematous or psoriasiform dermatoses) and are most often distributed on the trunk, especially the flanks, groin, and peri-axillary regions [8]. In the present case, the 37-year-old patient’s erythematous annular plaques with fading centers on the torso and groin closely mirrored these classic clinical features, reinforcing the diagnosis of ALDY.

Histopathology is pivotal in confirming ALDY and shows highly characteristic changes. A band-like (lichenoid) lymphocytic infiltrate is present at the dermoepidermal junction, with a striking pattern of keratinocyte injury: massive basal cell apoptosis confined to the tips of the rete ridges, imparting a squared (“quadrangular”) shape to the rete ridges [8,9]. This “quadrangular rete ridge” alteration, essentially the result of interface damage concentrated at rete ridge bases, is considered a diagnostic clue for ALDY [9]. The epidermis often shows irregular acanthosis with alternating areas of thinning and the aforementioned squared-off rete ridges. Vacuolar interface changes with colloid body formation can be prominent, but unlike mycosis fungoides, atypical lymphocytes are absent, and epidermal lymphocyte invasion (epidermotropism) is minimal [8]. There is no significant dermal fibrosis or sclerosis, a feature which helps rule out morphea or lichen sclerosus in the clinical differential [10]. Immunohistochemically, the dermal infiltrate is composed of benign T-lymphocytes (usually CD3+, predominantly CD4+ helper memory T-cells) with smaller numbers of CD8+ T-cells, B-cells, and macrophages [11]. Interestingly, many of the lymphocytes that do enter the epidermis in ALDY are CD8+, whereas the lichenoid dermal infiltrate is often rich in CD4+ cells [11]. Importantly, T-cell receptor gene rearrangement studies in reported cases consistently show a polyclonal T-cell population, supporting the benign, inflammatory nature of this disorder [12]. In summary, the combination of annular clinical morphology with a lichenoid tissue reaction, especially the focal basal keratinocyte apoptosis at rete ridge tips, is the hallmark of ALDY and was clearly demonstrated in our patient’s biopsy, effectively securing the diagnosis.

Epidemiology and demographics

ALDY is uncommon, but, to the best of our knowledge, over 60 cases have been documented in the literature since its initial description [3]. It predominantly affects children and adolescents, which is reflected in the name “of youth”. The mean age at presentation is approximately 10-15 years, and most cases occur before the early 20s [13]. However, subsequent reports have expanded the known age range considerably [13]. Adult-onset cases are now recognized, with cases reported from as young as 2 years up to 79 years of age [13]. Notably, Cesinaro et al., in 2009, were the first to report ALDY in adults, describing four adult male patients in their series (alongside two pediatric cases) and suggesting that the condition be renamed to “annular lichenoid dermatitis” as it is not exclusive to the young [14]. Our 37-year-old male patient firmly falls into this adult-onset category. The clinical and histologic phenotype in adult cases appears similar to that in children, although adults may pose a greater diagnostic challenge as clinicians more readily suspect MF or other diagnoses in an older patient (discussed below). There may be a slight male predominance overall (approximately 1.5:1 male-to-female ratio), and most patients are otherwise healthy, with no significant medical history or triggers identified [15].

In terms of geographic and ethnic distribution, ALDY cases have been reported from various parts of the world, but with a concentration in certain regions. Many early cases were from Mediterranean countries (the original series was from Italy) [13], and additional cases have come from Europe, the United States, and East Asia (Japan and Korea) [13]. There have also been reports from South Asia (e.g., India) [13]. To our knowledge, there have been no published cases from the Arabian Gulf region prior to this report, making our case the first from Kuwait. One brief report from Iran (a Middle Eastern country outside the Gulf) documented ALDY in two adults and one child [15], but otherwise the Middle East has been conspicuously absent in the ALDY literature. This lack of regional reports may be due to either true rarity or under-recognition of the entity [16]. Our case thus expands the epidemiologic spectrum of ALDY, indicating that it can occur in Middle Eastern populations and in older adults, and underscores the importance of considering this diagnosis even outside its initially described demographic group.

Differential diagnosis

The annular configuration and histologic lichenoid interface pattern in ALDY mandate careful consideration of several differential diagnoses. Given the implications of some look-alike conditions (e.g., cutaneous T-cell lymphoma), distinguishing ALDY from these differentials is critical for proper management. Key entities to consider are described below.

Mycosis Fungoides, Patch Stage

MF can present with annular or oval patches, sometimes with faint erythematous borders and central pale areas, especially in its hypopigmented variant seen in children [9,12]. Clinically, MF patches are often scaly or at least dry, and may be pruritic, whereas ALDY lesions tend to have a smoother surface and patients often lack pruritus. Histologically, MF is characterized by an epidermotropic infiltrate of atypical T-cells with cerebriform nuclei; one may see Pautrier’s microabscesses and alignment of atypical lymphocytes along the basal layer (tagging) with focal epidermal invasion [9,12]. In contrast, ALDY’s lymphocytic infiltrate, while also superficial, is lichenoid (dense and contiguous at the interface) and composed of benign-appearing lymphocytes [8,9]. Significantly, ALDY shows no atypical epidermal lymphocytes or convoluted nuclei, and very little epidermotropism [17]. Instead of the pervasive basal cell tagging seen in MF, ALDY has the focal rete ridge tip apoptosis pattern (absent in MF) [8,9,13]. Immunophenotyping can provide further clues: classic MF is usually a CD4+ T-cell lymphoma, though the hypopigmented form often has a CD8+ predominance; ALDY lesions typically demonstrate a mixture of CD4 and CD8 T-cells but with polyclonal TCR gene rearrangement, not a monoclonal expansion [11]. In difficult cases, gene rearrangement studies help: ALDY has consistently shown polyclonal T-cells, whereas MF generally reveals a clonal T-cell population in lesional skin. Clinically, ALDY does not progress to tumor stage or systemic involvement, unlike MF [8,14]. These distinctions are essential because misdiagnosing ALDY as MF could lead to overtreatment, while missing MF could be dangerous. In our adult patient, the concern for patch-stage MF was initially raised, but the absence of atypia and the presence of the characteristic squared rete ridge interface changes on biopsy favored ALDY over MF.

Sarcoidosis (Annular Cutaneous Sarcoidosis)

Granulomatous dermatoses such as cutaneous sarcoidosis or granuloma annulare can produce annular plaques with peripheral coloring and central clearing that mimic ALDY clinically [2]. Annular sarcoidosis typically shows red-brown annular plaques often on the trunk or face [2]. However, histopathology readily differentiates these conditions. Sarcoidosis shows collections of dermal epithelioid granulomas (often naked granulomas with little surrounding lymphocytic infiltrate), and it lacks the prominent basal cell damage seen in ALDY. There is no interface dermatitis in sarcoidosis; the epidermis in sarcoid lesions is usually unremarkable or only mildly acanthotic, without basal vacuolization or apoptotic keratinocytes [2]. In ALDY, in contrast, granulomas are absent, and the inflammatory process is centered at the dermoepidermal junction [8,13]. Additionally, sarcoid lesions tend to be more indurated (due to dermal granulomas and fibrosis), whereas ALDY patches are non-indurated. Our patient’s biopsy showed a lichenoid lymphocytic infiltrate with interface changes and no granulomas, effectively ruling out cutaneous sarcoidosis.

Eczematous Dermatitis (Nummular Eczema or Other Types)

Chronic eczematous processes can occasionally assume an annular configuration (e.g., nummular dermatitis may clear centrally or gyrate), prompting consideration of eczema in an annular plaque differential [2]. Unlike the asymptomatic lesions of ALDY, eczematous patches are often intensely itchy. Clinically, eczema lesions usually exhibit surface changes such as scale, crust, or lichenification, which were absent in our case [2]. Histologically, eczematous dermatitis is characterized by spongiosis (intercellular epidermal edema), sometimes with parakeratosis or serous crust and a mixed inflammatory infiltrate including lymphocytes and eosinophils [2]. While chronic eczema can show some degree of interface change or basal cell damage, it does not typically demonstrate the sharply localized basal keratinocyte apoptosis at rete tips nor the clean band-like infiltrate that defines ALDY [8,13]. The presence of abundant spongiosis or epidermal hyperkeratosis would point towards an eczematous process rather than ALDY. In our patient, the biopsy lacked spongiosis or significant scale, making eczema unlikely.

Therapeutic approaches and outcomes

There is no established definitive cure for ALDY, but various treatments have been tried with generally moderate success. ALDY tends to run a chronic, relapsing course, yet it is benign and often relatively asymptomatic. As a result, therapy is directed at accelerating lesion resolution and improving cosmetic appearance or patient comfort. Topical corticosteroids are the most commonly employed first-line treatment. High-potency topical steroids can reduce the lichenoid inflammation and often lead to partial or complete clearing of lesions over several weeks [17]. In many reports, lesions improved with topical steroid application, but it has also been noted that they tend to recur once treatment is tapered or stopped [1,17]. Our patient was started on potent topical corticosteroids, which led to significant flattening and fading of his plaques; however, consistent with the literature, new rings appeared when the steroid was discontinued, indicating a need for longer term maintenance.

Topical calcineurin inhibitors such as tacrolimus and pimecrolimus have also been reported to be effective in ALDY. These agents can be used especially on sensitive skin areas (like the groin) to avoid steroid atrophy and address T-cell-mediated inflammation. In one pediatric case, pimecrolimus cream achieved initial lesion clearance, although the dermatitis recurred after the medication was stopped, as with steroids. Tacrolimus ointment has been cited as one of the most effective therapies in reviews [14], and some authors favor it for long-term management given ALDY’s chronic nature and the need to minimize steroid use. In our case, adjunctive tacrolimus 0.1% ointment was utilized intermittently in rotation with a topical steroid, resulting in good control of active lesions.

Phototherapy is another therapeutic modality that has been employed for ALDY, with varying outcomes. Ultraviolet light (narrowband UVB or PUVA) can have a T-cell-modulating effect, similar to its use in early mycosis fungoides. Annessi et al. reported that phototherapy (e.g., narrowband UVB) was effective in inducing remission in most of their patients [1]. However, other case reports note only partial response or relapse after phototherapy. For instance, Stojkovic-Filipovic et al. described a child in whom UVB phototherapy, combined with topical steroids, failed to produce improvement [17]. In our adult patient, we tried a course of narrowband UVB, given the extensive trunk involvement; this yielded some transient improvement in pigmentation, but the annular lesions persisted, echoing the mixed results seen in the literature.

Refractory or widespread ALDY has been occasionally managed with systemic therapy. Short courses of systemic corticosteroids have been used in severe cases to achieve quicker control of extensive lesions, but lesions may reappear as the systemic steroids are tapered off. More recently, cyclosporine (an immunosuppressant targeting T-cells) was reported to induce a complete remission in a child with recalcitrant ALDY [17]. In that case, prior therapies (including antihistamines, topical steroids, and phototherapy) had failed, but low-dose cyclosporine led to clearance of lesions within weeks. This suggests that ALDY’s inflammation is T-cell driven and can be effectively dampened by systemic immunosuppression in aggressive cases. Other immunomodulators or biologics have not yet been systematically reported for ALDY, likely because most patients ultimately do well with topical therapies and the disease does not threaten systemic health.

The prognosis of ALDY is generally favorable. ALDY is a benign condition with no malignant potential; importantly, long-term follow-up of patients has not shown evolution into mycosis fungoides or other lymphoproliferative disorders. Many patients experience a chronic course with periods of remission and relapse, but some may undergo spontaneous resolution of lesions over time. Cesinaro et al. documented complete resolution of ALDY lesions in four of six patients after prolonged follow-up of two to five years [14]. It is postulated that the dermatosis may eventually “burn out” in some individuals. In our patient’s case, after a year of on-and-off therapy, the lesions have significantly diminished in number and intensity, and no new lesions have appeared in recent months. This outcome aligns with the notion that ALDY, while persistent, can gradually abate.

## Conclusions

In conclusion, management of ALDY should be individualized, focusing on anti-inflammatory therapies and long-term monitoring. Topical corticosteroids and calcineurin inhibitors are first-line and can be used safely for extended periods, given the superficial nature of the disease. Phototherapy can be considered for more extensive cases, although responses are variable. Systemic agents like cyclosporine are reserved for severe, unresponsive cases. Patient education is important to set expectations: ALDY lesions may recur when treatment stops, and the condition can wax and wane before eventual resolution. Our report, which, to the best of our knowledge, is the first in Kuwait and the Gulf region, highlights that recognition of ALDY in adults is important to avoid misdiagnosis (such as MF) and to guide appropriate therapy. With awareness of its distinct clinicopathological features and benign course, clinicians can spare patients from overly aggressive treatments and instead employ targeted therapies that achieve disease control with minimal side effects.
